# Efferocytosis: The Janus‐Faced Gatekeeper of Aging and Tumor Fate

**DOI:** 10.1111/acel.14467

**Published:** 2025-01-03

**Authors:** Zaoqu Liu, Yan Li, Yuqing Ren, Jingqi Chen, Siyuan Weng, Zhaokai Zhou, Peng Luo, Quan Chen, Hui Xu, Yuhao Ba, Anning Zuo, Shutong Liu, Yuyuan Zhang, Teng Pan, Xinwei Han

**Affiliations:** ^1^ Department of Interventional Radiology The First Affiliated Hospital of Zhengzhou University Zhengzhou Henan China; ^2^ Interventional Institute of Zhengzhou University Zhengzhou Henan China; ^3^ Interventional Treatment and Clinical Research Center of Henan Province Zhengzhou Henan China; ^4^ Institute of Basic Medical Sciences Chinese Academy of Medical Sciences and Peking Union Medical College Beijing China; ^5^ Medical School of Zhengzhou University Zhengzhou Henan China; ^6^ Department of Respiratory and Critical Care Medicine The First Affiliated Hospital of Zhengzhou University Zhengzhou Henan China; ^7^ Department of Urology The First Affiliated Hospital of Zhengzhou University Zhengzhou Henan China; ^8^ The Department of Oncology, Zhujiang Hospital Southern Medical University Guangzhou China; ^9^ Department of Neurosurgery, Xiangya Hospital Central South University Changsha Hunan China; ^10^ Longgang District Maternity & Child Healthcare Hospital of Shenzhen City (Longgang Maternity and Child Institute of Shantou University Medical College) Shenzhen Guangdong China

**Keywords:** aging, efferocytosis, inflammation, tumor immunity

## Abstract

From embryogenesis to aging, billions of cells perish daily in mammals. The multistep process by which phagocytes engulf these deceased cells without eliciting an inflammatory response is called efferocytosis. Despite significant insights into the fundamental mechanisms of efferocytosis, its implications in disorders such as aging and cancer remain elusive. Upon summarizing and analyzing existing studies on efferocytosis, it becomes evident that efferocytosis is our friend in resolving inflammation, yet it transforms into our foe by facilitating tumor development and metastasis. This review illuminates recent discoveries regarding the emerging mechanisms of efferocytosis in clearing apoptotic cells, explores its connections with aging, examines its influence on tumor development and metastasis, and identifies the regulatory factors of efferocytosis within the tumor microenvironment. A comprehensive understanding of these efferocytosis facets offers insights into crucial physiological and pathophysiological processes, paving the way for innovative therapeutic approaches to combat aging and cancer.

## Graphical abstract

The multistep process by which phagocytes engulf these deceased cells without eliciting an inflammatory response is called efferocytosis. During aging, the efferocytosis of phagocytes is impaired, which is closely associated with the development of many age‐related diseases. The large accumulation of senescent cells in the body prevents phagocytosis from removing dead cells, which promotes chronic tissue inflammation. Additionally, efferocytosis promotes tumor growth and metastasis by inhibiting inflammatory responses and antitumor immunity.

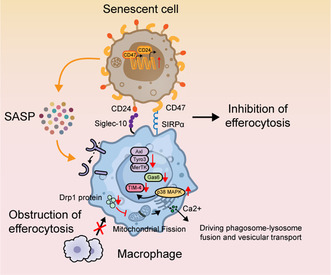

**Abbreviations**
AMPK: adenosine monophosphate‐activated protein kinase
ATP: adenosine triphosphate
BAI1: brain‐specific angiogenesis inhibitor 1
CHI3L1: chitinase 3‐like protein 1
CRT: calreticulin
CX3CL1: C‐X3‐C motif chemokine ligand 1
DNMT3A: DNA methyltransferase‐3A
DOCK: dedicator of cytokinesis
Drp1: dynamin‐related protein 1
ECM: extracellular matrix
EGF: epidermal growth factor
EIMP: efferocytosis‐induced macrophage proliferation
ELMO: engulfment and cell mobility
EPO: erythropoietin
ERK1/2: extracellular signal‐regulated kinase 1/2
FADD: Fas‐associated death domain protein
FGF: fibroblast growth factor
Gas6: growth arrest‐specific protein 6
Klf4: Krüppel‐like factor 4
LAP: LC3‐associated phagocytosis
LC3: microtubule‐associated protein 1A/1B light chain 3
LPC: lysophosphatidylcholine
MAPK: mitogen‐activated protein kinase
MBL: mannose‐binding lectin
MFG‐E8: milk fat globule–epidermal growth factor 8
MMP‐9: matrix metallopeptidase‐9
NET: neutrophil extracellular trap
NRF1: nuclear respiratory factor 1
PD‐L1: programmed cell death 1 ligand 1
PI3K: phosphatidylinositol 3‐kinase
PPAR: peroxisome proliferator‐activated receptor
PS: phosphatidylserine
Ptgs2: prostaglandin‐endoperoxide synthase 2
Rac1: Ras‐related C3 botulinum toxin substrate 1
SASP: senescence‐associated secretory phenotype
SHIP1: Sh2‐containing inositol 5′‐phosphatase 1
SIRPα: signal‐regulatory protein‐α
SIGLEC10: sialic acid‐binding Ig‐like lectin 10
SOCS1/3: suppressor of cytokine signaling 1/3
S1P: sphingosine‐1‐phosphate
TAMs: tumor‐associated macrophages
TIM1: T cell immunoglobulin mucin receptor 1
VEGF: vascular endothelial growth factor

## 1 Introduction

In most physiological and pathological scenarios, cells die through a series of programmed signaling events (Galluzzi et al. 2018) and are replaced by new cells derived from stem cell progenitors (Bergmann and Steller 2010). In human physiology, cell death is closely related to development and aging (Suzanne and Steller 2013; Tower 2015). During human growth and development, billions of cells are eliminated to form new tissue structures (Suzanne and Steller 2013). Meanwhile, mis‐regulation of cell death is increasingly implicated in aging (Tower 2015). In the course of human pathology, tissue damage, infections, and inflammatory responses lead to massive cell death. Therefore, to maintain homeostasis, dead cells must be effectively removed. The multistep process by which phagocytes engulf and remove dying cells without triggering an inflammatory response is known as efferocytosis. Efferocytosis is similar to entosis in that it is a “cell‐eats‐cell” phenomenon, except that efferocytosis involves phagocytes actively recognizing and engulfing different types of dead or dying cells, whereas entosis is a process in which the internalized cell itself penetrates the host cell, and it occurs only between homotypic cells (Gaptulbarova et al. 2024).

Existing research suggests that efferocytosis plays an active role in tissue recovery, inflammation reduction, and immune system homeostasis (Ge, Huang, and Yao 2022). Impaired efferocytosis results in impaired clearance of dying cells, accumulation of dead cells, cell necrosis and cytolysis, release of intracellular contents into the surrounding environment, and activation of inflammatory immune pathways (Arandjelovic and Ravichandran 2015; Szondy et al. 2014). These unfavorable outcomes can harm surrounding healthy cells and tissues. Meanwhile, a significant inflammatory response and the emergence of several inflammatory illnesses may be caused by aberrant efferocytosis (Doran, Yurdagul, and Tabas 2019). Although efferocytosis is our friend when it comes to addressing inflammation, it plays the role of a foe in tumor disease (Lin et al. 2020). Efferocytosis shapes the immunosuppressive microenvironment, helping tumors evade antitumor immunity and promoting tumor cell metastasis (Vaught, Stanford, and Cook 2015).

In what follows, we have combed through and summarized some of the most recent research advances on efferocytosis, including the mechanism of efferocytosis, the suppression of inflammation by efferocytosis (focusing primarily on the relationship between efferocytosis and chronic inflammation in aging), the role of efferocytosis in tumor progression and metastasis, and the factors that regulate efferocytosis in the tumor microenvironment. Among them, an interesting finding is that efferocytosis is inhibited during aging, so the clearance of aging cells and dead cells in the body slows down, which may be one of the reasons for the low level of chronic inflammation in the body during aging. In addition, we found that efferocytosis can promote the growth and metastasis of established tumors but also can control the development of inflammatory precancerous lesions into cancer. Understanding these aspects of efferocytosis is helpful to put forward novel therapeutic strategies for aging and tumor diseases.

## 2 Emerging Mechanisms of Efferocytosis

**FIGURE 1:**
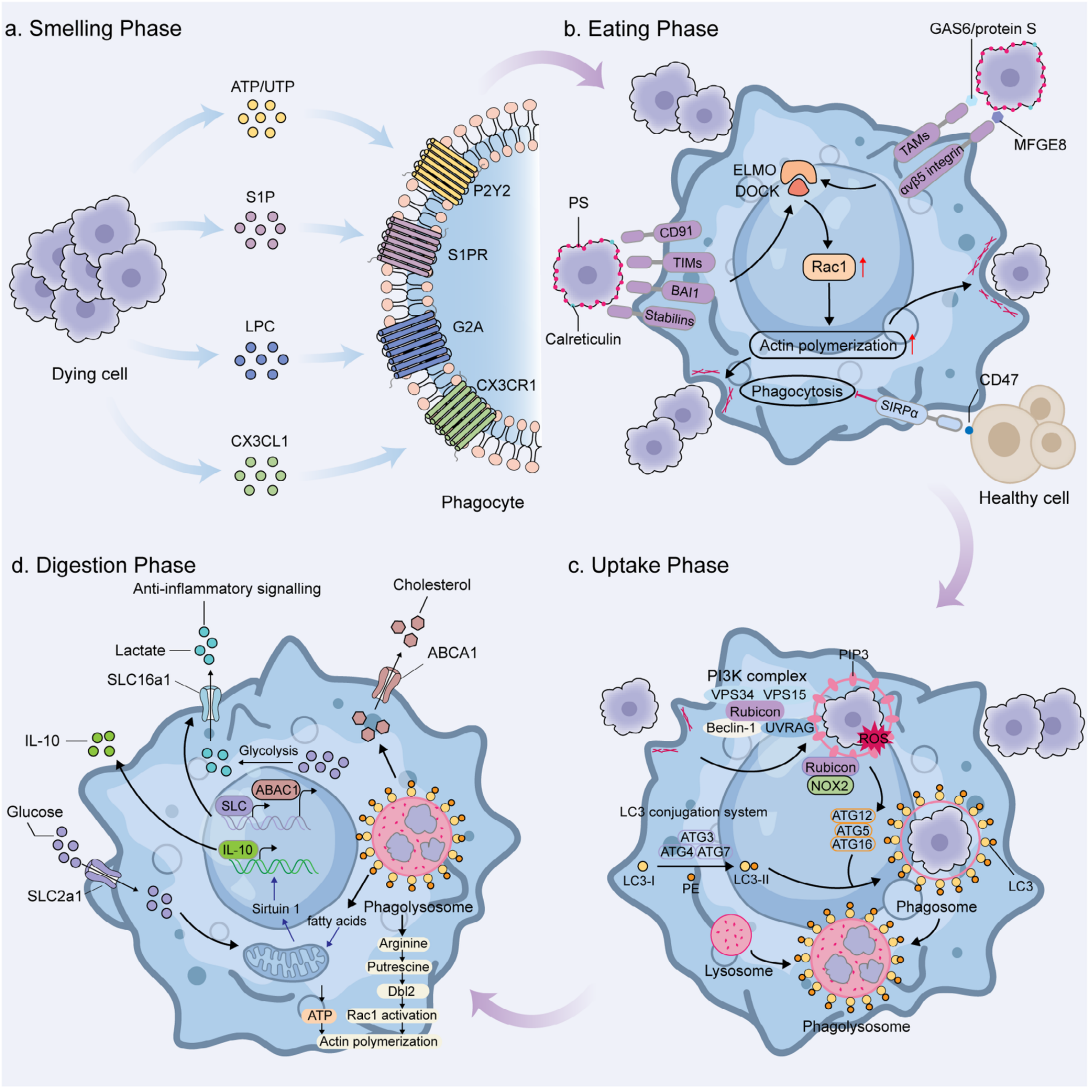
The process of efferocytosis. (a) Smelling phase: A series of “find‐me” signaling molecules including ATP, UTP, CX3CL1, S1P, and LPC are released by dying cells to initiate the process of efferocytosis. These signaling molecules are recognized by phagocytes through several surface receptors and attract phagocytes to the area where dying cells are located. (b) Eating phase: A primary “eat‐me” signal exposed on dying cells is externalized PS, which can interact with a variety of receptors on the surface of the phagocyte, including TIMs, BAI1, and stabilins. Also, PS can be bound by phagocytes through receptors, including MERTK, via adaptors including GAS6 and protein S, or through avβ5 integrin via MFG‐E8. Calreticulin can also act as an “eat‐me” signal through binding to CD91 expressed on the phagocyte outer membrane. Key downstream efferocytic signaling involves GTPase RAC1, which is activated via ELMO and DOCK proteins to enhance actin polymerization and formation of the phagocytic cup. CD47 is the classic “don't‐eat‐me” signal expressed by healthy cells and recognized by SIRPα on the surface of phagocytes, preventing phagocytosis from occurring. (c) Uptake phase: Upon internalization of dying cells, phagosomes recruit Rubicon and induce assembly of the PI3K complex. Activation of the LC3 conjugation system leads to lipidation of LC3 and its localization to phagosomes. Subsequently the phagosome fuses with the lysosome to form a phagolysosome. (d) Digestion phase: Lysosomal degradation of dying cell releases free fatty acids, cholesterol, amino acids, sugars, and nucleic acids. Of these, arginine is converted to putrescine by ornithine decarboxylase, which activates Rac1 and drives subsequent efferocytosis. Cholesterol is transported outside the cell via ABCA1 transporter proteins that are upregulated during efferocytosis. Additionally, efferocytosis leads to solute carrier family reprogramming, resulting in upregulation of SLC2A1 and SLC16a1. Enhanced glucose input leads to increased glycolysis and increased ATP production, which promotes actin polymerization. Lactate, which is the result of increased aerobic glycolysis, is secreted extracellularly via SLC16a1 and acts as an anti‐inflammatory mediator. Fatty acids activate sirtuin1 signaling, which then triggers expression of IL‐10.

In general, the process of efferocytosis ends apoptosis, preventing the accumulation of dead cells, inflammation, and subsequent cell necrosis (Poon et al. 2014). Specifically, efferocytosis involves four steps: (1) dying cells recruit phagocytes with “find‐me” signals (Ravichandran 2003), (2) “eat‐me” and “don't‐eat‐me” signals guide phagocytes to recognize dead and non‐dead cells (Segawa and Nagata 2015), (3) phagocytes ingest cellular corpses, and (4) dying cells are degraded in phagocytes (Figure 1). To guarantee accurate and effective evacuation of dying cells, each of these processes is strictly controlled.

### 2.1 The Smelling Phase

The apoptotic cells recruit macrophages by releasing soluble signals into the environment. These signals are known as “find‐me” signals, which include lysophosphatidylcholine (LPC) (Lauber et al. 2003), sphingosine‐1‐phosphate (S1P), ATP, UTP (Elliott et al. 2009), and C‐X3‐C motif chemokine ligand 1 (CX3CL1) (Truman et al. 2008) (Table 1). The S1P mitogen‐activated protein kinases SPK1 and SPK2, which phosphorylate the membrane lipid sphingosine to create S1P, are upregulated in apoptotic cells (Gude et al. 2008; Weigert et al. 2006). As well, caspase 3 cleavage activates phospholipase A2, allowing LPC to be synthesized from phosphatidylcholine (Lauber et al. 2003). Nucleotides such as ATP and UTP facilitate efferocytosis by prompting phagocytosis to contact purinergic receptors (Elliott et al. 2009). Furthermore, through its interaction with the CX3CR1, the chemokine CX3CL1 draws macrophages to the location of apoptotic cells (Truman et al. 2008). In recent years, it has been found that neutrophils can also exert efferocytosis. The “find‐me” signals released by apoptotic cells that attract neutrophils include CCL3, CXCL1, CXCL5, CXCL8/IL8, tyrosyl tRNA synthetase (TyrRS), and endothelial monocyte activating polypeptide II (EMAPII) (Cullen et al. 2013; Ramos and Oehler 2024; Schimek et al. 2022).

**TABLE 1:** Summary of signaling molecules and their receptors in efferocytosis.

Signal	Molecule	Receptor
Find me	S1P	S1PR
	LPC	G2A
	ATP/UTP	P2Y2
	CX3CL1	CX3CR1
Eat me	PS	Direct binding
		TIM1, TIM4, BAI1, RAGE, CD300f, stabilin 1/2
		Indirect binding
		MERTK, avβ5 integrin
	Calreticulin	CD91
Don't eat me	CD47	SIRPα
	CD24	SIGLEC10
	CD31	CD31

Abbreviations: G2A, G protein‐coupled receptor; LPC, lysophosphatidylcholine; PS, phosphatidylserine; S1PR, sphingosine‐1‐phosphate receptor; SIRPα, signal‐regulatory protein‐α.

Furthermore, when ‘find‐me’ signals bind to their appropriate receptors, they can alter phagocyte behavior. For instance, CX3CL1 stimulates the expression of milk fat globule–epidermal growth factor 8 (MFG‐E8) by phagocytes, which bridges apoptotic cells to the phagocytes to facilitate engulfment (Miksa et al. 2007). S1P binding to the S1P receptor on macrophages promotes macrophage erythropoietin (EPO) signaling, and EPO can enhance macrophage efferocytosis via peroxisome proliferator‐activated receptor‐γ (PPAR‐γ) (Luo et al. 2016).

### 2.2 The Eating Phase

In this phase, the ‘eat‐me’ signals exposed on the surface of apoptotic cells bind directly or indirectly to receptors on the surface of phagocytes. These signals allow phagocytes to distinguish between apoptotic cells and surrounding healthy cells.

The most well‐known and classic “eat‐me” signal among the newly appeared molecules on the surface of dying cells is phosphatidylserine (PS). In apoptotic cells, PS is translocated from the inner surface to the outer surface of the plasma membrane (Fadok et al. 1998), where it binds directly to phagocyte surface receptors, such as T cell immunoglobulin mucin receptor 1 (TIM1), TIM4, brain‐specific angiogenesis inhibitor 1 (BAI1), stabilin 2, and members of the CD300 family (Table 1) Alternatively, phagocyte surface receptors such as integrins and tumor‐associated macrophage (TAM) receptors might bind to PS indirectly through the mediation of soluble bridging proteins (Geng et al. 2017). It is worth mentioning that PS exposed on the outer cell surface is not only an “eat‐me” signal for apoptotic cell efferocytosis but also participates in blood coagulation, myoblast fusion, and immunomodulation of non‐apoptotic cells (Chang et al. 2020; Elliott et al. 2005; Srivasatava et al. 2014; van den Eijnde et al. 2001).

Although PS is a strong and well‐known “eat‐me” signal, other signals may also be involved in the identification and clearance of dying cells. LPCs exposed on the surface of dying cells are able to bind to IgM, which then binds to the Fc receptors of phagocytes (Kim et al. 2002). Therefore, LPC seems to be a signal for both finding and eating. Additionally, calreticulin (CRT) can also be exposed to the plasma membrane of dying cells when apoptosis occurs, acting as an “eat‐me” signal (Gardai et al. 2005). Phagocytes recognize CRT via CD91 in concert with complement C1q and mannose‐binding lectin (MBL) (Ogden et al. 2001).

Although living cells generally do not express “eat‐me” signals, some living cells transiently express markers that partially mimic dying cells under specific physiological circumstances, such as during lymphocyte activation, skeletal muscle formation, and sperm–egg fusion (Kelley and Ravichandran 2021). Thus, expression of “don't eat‐me” signaling prevents the unwarranted removal of “healthy” cells. CD47 and CD24 are two well‐known “don't‐eat‐me” signals that bind to signal‐regulatory protein‐α (SIRPα) and sialic acid binding Ig like lectin 10 (SIGLEC10), respectively, on macrophages (Barkal et al. 2019; Oldenborg et al. 2000). In addition, it is worth mentioning that cancer cells can evade macrophage clearance by overexpressing the “don't eat‐me” signal (Barkal et al. 2019).

### 2.3 The Uptake and Digestion Phase

Once phagocytes recognize dying cells, phagocytes undergo rapid plasma membrane reorganization and synthesis in order to efficiently engulf the dying cells (Richards and Endres 2014). Phagocyte plasma membrane shape changes require the involvement of a dynamic meshwork of actin fibers located beneath the plasma membrane. Receptor–ligand interactions stimulate the binding of engulfment and cell mobility (ELMO) proteins to dedicator of cytokinesis 180 (DOCK180) in phagocytes to form a complex that activates the Ras‐related C3 botulinum toxin substrate 1 (Rac‐1) signaling pathway (Gumienny et al. 2001; Park et al. 2007). This causes cytoskeletal rearrangements and internalization of apoptotic cells, leading to the formation of phagosomes. Internalization of apoptotic cells drives the assembly of the phosphatidylinositol 3‐kinase (PI3K) complex in phagocytes, which includes Rubicon, UVRAG, Beclin 1, VPS34, and VPS15 (Martinez et al. 2015). This complex promotes microtubule‐associated protein 1A/1B light chain 3 (LC3) proteins binding to phagosomal lipid membranes. This process, termed LC3‐associated phagocytosis (LAP), promotes not only phagosome maturation but also phagosome–lysosome fusion, ultimately increasing efferocytosis efficiency (Martinez et al. 2011). Phagosomes fuse with lysosomes to form phagolysosomes, in which large amounts of proteases, nucleases, and lipases degrade fragments of dying cells (Boada‐Romero et al. 2020).

Inside the phagocyte, substances produced by the digestion of dying cells need to be metabolized and then reentered into the metabolic cycle of the phagocyte or excreted from the cell. Meanwhile, these metabolites may affect the subsequent efferocytosis of phagocytes. For example, it has been shown that macrophages utilize arginine and ornithine produced by metabolizing apoptotic cells to facilitate subsequent efferocytosis (Yurdagul et al. 2020) (Figure 1).

## 3 Efferocytosis Is the Firefighter of Inflammation, Whereas Impaired Efferocytosis Is the Propellant of Inflammation

**FIGURE 2:**
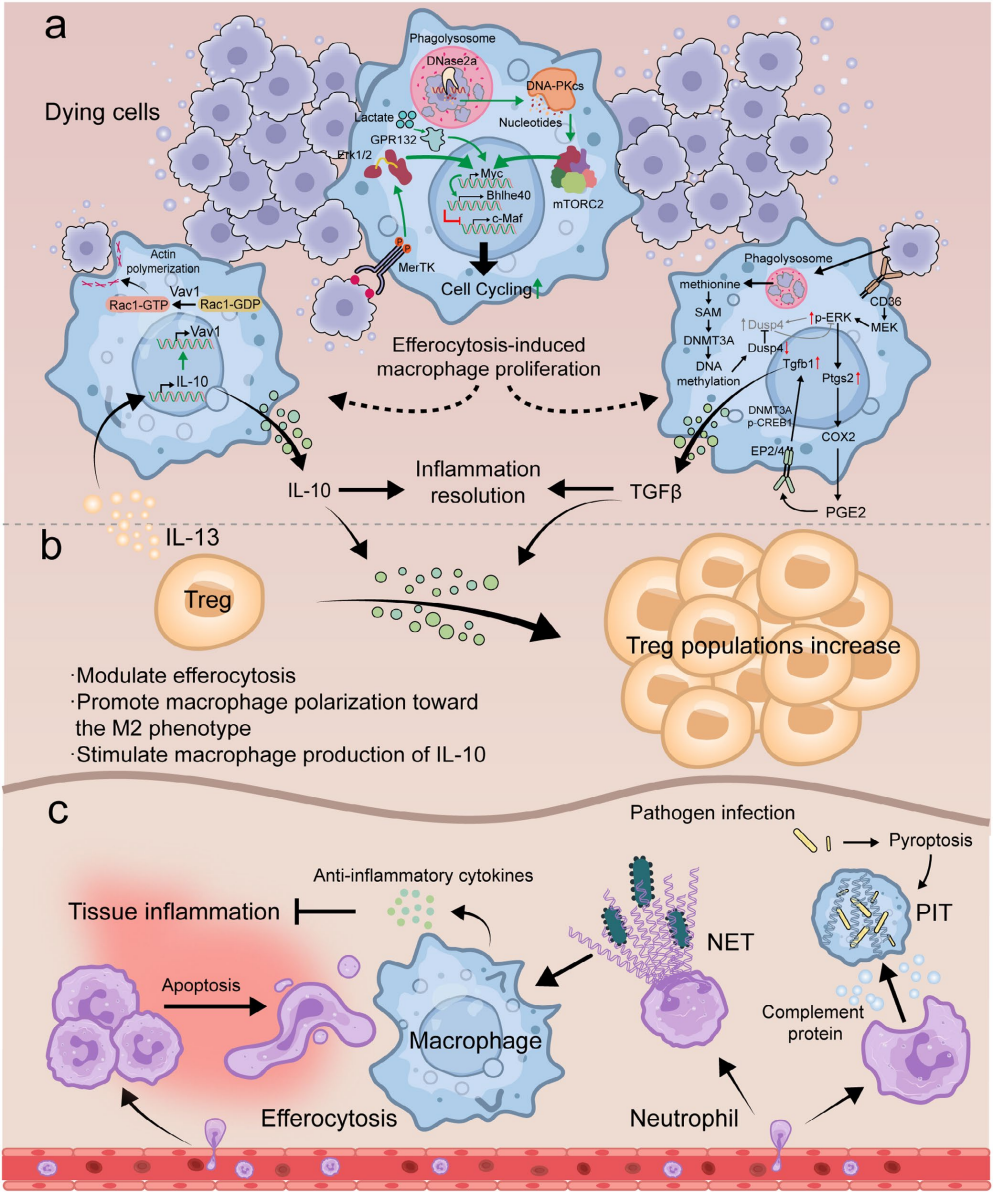
Efferocytosis in immune cells. Efferocytosis is involved in the body's anti‐infective immunity and suppression of inflammation development. It is closely related to immune cells such as macrophages, neutrophils, and Treg. (a) Macrophages are the primary cells that exercise efferocytosis. They ingest and degrade dying cells, limiting cell necrosis. Meanwhile, efferocytosis also has an effect on macrophages. First, efferocytosis induces macrophage proliferation, a process realized by two independent pathways. In the first pathway, DNase2a in the phagolysosomes formed by macrophages after ingestion of dying cells hydrolyzes DNA from dying cells to produce nucleotides. The nucleotides activate the DNA–PKcs–mTORC2 pathway, which increases the pro‐proliferative transcription factor Myc. Myc upregulates Bhlhe40 and downregulates c‐Maf, thereby promoting proliferation of non‐inflammatory macrophages. This pathway also requires additional stimulation by MerTK‐ERK1/2. The second pathway is efferocytosis‐induced production of lactate that promotes Myc protein stabilization via GPR132. In addition, macrophages can utilize methionine produced by degraded dying cells to promote tissue inflammation reduction. Activation of CD36 by dying cells induces ERK phosphorylation, which activates the Ptgs2–PGE2–TGF‐β pathway. However, the ERK–Dusp4 negative feedback pathway reduces ERK phosphorylation. Fortunately, methionine produced during efferocytosis is converted to SAM, which is used by DNMT3A to methylate Dusp4. As a result, Dusp4 is inhibited and the Ptgs2–PGE2–TGF‐β pathway is enhanced. (b) Treg is involved in the modulation of efferocytosis. IL‐13 secreted by Treg stimulates macrophages to secrete IL‐10, which induces an increase in the expression of Vav1 in macrophages. Vav1 activates Rac1, which in turn leads to actin rearrangement within the macrophage cell membrane and enhances efferocytosis. In addition, TGF‐β and IL‐10 secreted by macrophages can increase Treg population. (c) When tissue is infected or injured, neutrophils are recruited from the vasculature to the site of inflammation. Large numbers of neutrophils accumulate at the site of inflammation, and some of these neutrophils undergo apoptosis. Macrophages remove apoptotic neutrophils through efferocytosis and secrete anti‐inflammatory cytokines to limit the development of tissue inflammation. In addition, when pathogens invade, neutrophils release NETs to capture the pathogens. NETs can eventually be phagocytosed and cleared by macrophages. Alternatively, neutrophils can also exert efferocytosis. Pathogen infection induces macrophages to undergo pyroptosis. Pyroptotic macrophages form PIT to capture bacteria. Complement mediates neutrophil recruitment to the PIT site. Subsequently, neutrophils clear the PIT through efferocytosis.

In general, controlled inflammation contributes to the clearance of invasive pathogens, the removal of injured cells, and the initiation of the wound‐healing response, but it can become detrimental if dysregulated. Efferocytosis removes dead cells, contributing to the resolution of inflammation. In inflammation, inflammatory cells (e.g., neutrophils, lymphocytes) may undergo programmed cell death after functioning and are subsequently phagocytosed and cleared by macrophages through efferocytosis (Sendama 2020). This specific form of phagocytosis by macrophages prevents inflammatory cells from undergoing secondary necrosis and thus protects surrounding tissues from damage by tissue‐toxic cellular contents (Rydell‐Törmänen, Uller, and Erjefält 2006). Meanwhile, efferocytosis can reprogram macrophages to promote inflammation resolution and facilitate tissue repair (Huynh, Fadok, and Henson 2002). Inflammatory immune cells that are closely associated with efferocytosis include macrophages, neutrophils, and regulatory T cells. They can be removed by efferocytosis and play unique roles in the efferocytosis process (Figure 2).

Furthermore, under physiologic conditions, normal efferocytosis does not induce an inflammatory response; however, when efferocytosis is inhibited or impaired, it can lead to inflammatory diseases. A great example of this is the chronic inflammation present in aging. With aging, older adults are more susceptible to infections and prone to age‐related chronic diseases (Pawelec, Goldeck, and Derhovanessian 2014). Even in the absence of infection, older adults have been shown to have elevated levels of circulating pro‐inflammatory cytokines and acute‐phase proteins (Franceschi et al. 2018). Inhibition of efferocytosis during aging may be an important cause of inflammaging.

### 3.1 Efferocytosis and Immune Cells in Inflammation

#### 3.1.1 Efferocytosis and Macrophages

Macrophages are the predominant specialized phagocytes that exercise efferocytosis (Boada‐Romero et al. 2020). They uptake, degrade, and metabolize apoptotic cells and limit cell necrosis. Meanwhile, efferocytosis affects the proliferation and differentiation of macrophages.

Recently, it has been shown that efferocytosis induces macrophage proliferation (Gerlach et al. 2021; Ngai, Schilperoort, and Tabas 2023). Efferocytosis‐induced macrophage proliferation (EIMP) needs two pathways to work together in a coordinated manner: a signaling pathway induced by apoptotic cell‐derived nucleotides (Gerlach et al. 2021) and a cellular metabolism pathway involving lactate production (Ngai, Schilperoort, and Tabas 2023). In the first pathway, the DNA–PKcs–mTORC2/Rictor pathway is activated by nucleotides obtained from the breakdown of apoptotic cell DNA by phagolysosomal DNase2a, which raises Myc. Myc upregulates Bhlhe40 and downregulates c‐Maf to promote noninflammatory macrophage proliferation. This process requires additional stimulation from MerTK. In another pathway, efferocytosis induces the production of lactate. GPR132‐mediated lactate signaling encourages the stability of Myc protein and the subsequent proliferation of macrophages. This type of macrophage proliferation occurs in vivo in acute models of high‐burden efferocytosis (Park et al. 2011; Yurdagul et al. 2020).

Furthermore, efferocytosis induces macrophage polarization toward the M2 phenotype (Geng et al. 2022; Lin et al. 2022; Myers, Amend, and Pienta 2019). M2 macrophages tend to release more anti‐inflammatory mediators (IL‐10 and TGF‐β) (Angsana et al. 2016). After efferocytosis, the lipid components and metabolites of apoptotic cells bind to PPARs and LXRs. These nuclear receptors that have been activated function as transcription factors to increase the expression of genes linked to M2 phenotype (Bouhlel et al. 2007; Kimura et al. 2016; Penas et al. 2015). In addition, efferocytosis enhances the phosphorylation of extracellular signal‐regulated kinase (ERK) in macrophages and induces the Ptgs2–PGE2–TGF‐β1 pathway, which promotes the reduction of tissue inflammation (Ampomah et al. 2022) (Figure 2).

#### 3.1.2 Efferocytosis and Neutrophils With Neutrophil Extracellular Traps

When an infection or injury occurs, neutrophils are the first immune cells recruited to the site of inflammation. However, the inflammatory response is intensified by massive neutrophil aggregates and secondary necrosis, which results in more severe tissue damage and organ dysfunction. Fortunately, efferocytosis can improve this outcome. Apoptotic neutrophils can be cleared by macrophages through efferocytosis. Efferocytosis prevents neutrophil lysis and the subsequent release of cytotoxic and inflammatory components (Greenlee‐Wacker 2016). When external pathogens invade the body, neutrophils can capture, neutralize, and kill pathogens by phagocytosis or releasing neutrophil extracellular traps (NETs). NETs are net‐like chromatin structures secreted by activated neutrophils and are primarily composed of free deoxyribonucleic acid, histones, and granulin (Brinkmann et al. 2004; Papayannopoulos 2018). In vitro studies have demonstrated that NETs can also be taken up and degraded by macrophages through efferocytosis (Bukong et al. 2018), and that this process of NET clearance is immunosilenced (Farrera and Fadeel 2013). There was a lot of talk before that the primary mechanism known to be involved in NET elimination is extracellular DNase I degradation (Hakkim et al. 2010). However, DNase I alone will not be enough to remove NETs completely. It has been shown that NETs are cleared by phagocytosis of monocyte‐derived macrophages and DNase I can help break down NETs so that macrophages can more easily remove them afterward (Farrera and Fadeel 2013). Following uptake, degradation of NETs in macrophages is dependent on DNase‐III (TREX1) (Lazzaretto and Fadeel 2019). Moreover, macrophages do not produce pro‐inflammatory cytokines after NET clearance and do not cause an inflammatory response. However under physiological conditions, NET production is probably initiated by pathogens. Stimulation of macrophages by bacterial LPS together with NETs leads to pro‐inflammatory cytokine production.

Neutrophils, a type of phagocyte, can also remove infected cells through efferocytosis. After *Salmonella typhimurium* ‐infected macrophages undergo pyroptosis, the intracellular bacteria are surrounded by a largely intact plasma membrane and remain within the pyroptotic cell corpses. This structure in which pyroptotic cells encircle intracellular bacteria is known as the pore‐induced intracellular trap (PIT), which is conceptually parallel to the NET. In vivo, pyroptotic cell corpses and PITs are mainly taken up and removed by neutrophils through efferocytosis (Jorgensen et al. 2016).

#### 3.1.3 Efferocytosis and Other Immune Cells

Regulatory T cells (Tregs) promote macrophage polarization toward the M2 phenotype and release anti‐inflammatory mediators, which in turn enhance immune tolerance and accelerate inflammation resolution (Josefowicz, Lu, and Rudensky 2012; Weirather et al. 2014).

It has been found that regulatory T cells modulate efferocytosis in inflammation in animal models of peritonitis, lung injury, and advanced atherosclerosis (Proto et al. 2018). Depletion of Tregs reduces the efferocytic capacity of macrophages in tissues during the inflammatory regression phase of peritonitis and acute lung injury. In advanced atherosclerosis, there is impaired efferocytosis that promotes disease progression, whereas expanding Treg cell can improve efferocytosis (Proto et al. 2018). Tregs release IL‐13 to trigger the production of IL‐10 by macrophages. This, in turn, promotes the efferocytosis of macrophages by starting the accumulation of Rac1‐associated actin in phagosomes and the internalization of apoptotic cells (Proto et al. 2018). Macrophages produce IL‐10 and TGF‐β during efferocytosis, further increasing Treg populations (Kleinclauss et al. 2006).

### 3.2 The Subtle Relationship Between Inflammation, Aging, and Efferocytosis

**FIGURE 3:**
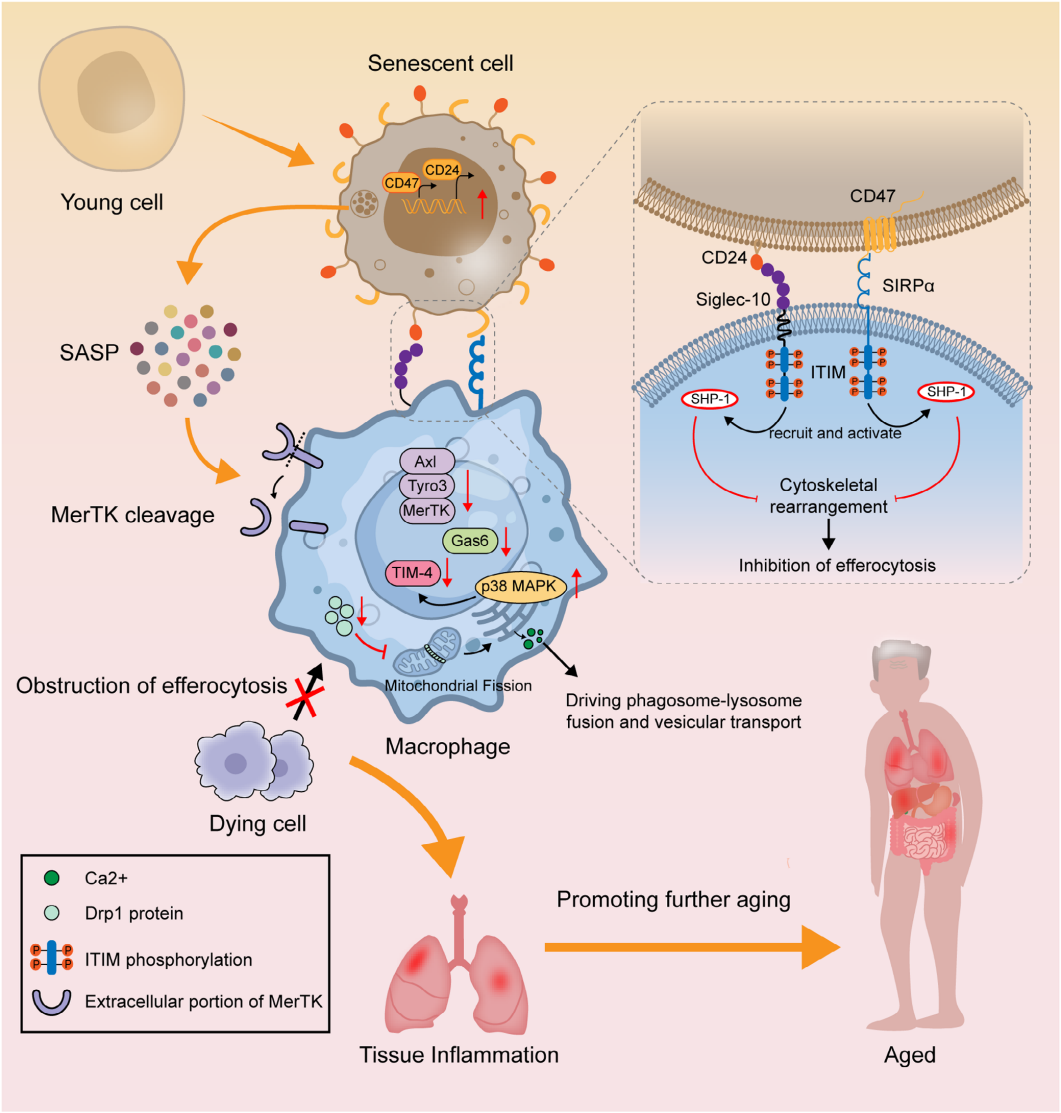
Impaired efferocytosis in aging. Senescent cells have increased expression of CD47 and CD24, which bind to SIRPα and Siglec‐10 on the macrophage surface, respectively. This leads to phosphorylation of ITIM structural domain proteins in macrophages, which results in recruitment and activation of SHP‐1. Activated SHP‐1 inhibits cytoskeletal rearrangements through downstream signaling pathways, thereby inhibiting efferocytosis. Furthermore, senescent cells secrete SASP to cleave MerTK on the macrophage surface, shedding its extracellular portion and thus preventing it from performing its normal function. In aging, macrophages have decreased expression of TAM receptors (including MerTK, Axl, and Tyro3) and Gas6, which impairs macrophage binding to dying cells. In addition, mitochondrial fission promotes Ca^2+^ release from the endoplasmic reticulum, driving phagosome–lysosome fusion and vesicular transport. However, Drp1 protein, a key protein involved in mitochondrial fission, has decreased activity in aging, leading to dysregulation of mitochondrial fission. The resultant combination of the above events leads to impaired efferocytosis in aging, resulting in tissue inflammation that promotes further aging.

Even in the absence of infection, circulating pro‐inflammatory cytokines and acute‐phase proteins are increased in older adults, suggesting the presence of a low level of chronic inflammation during the aging process. This phenomenon is called “inflammaging” (Franceschi et al. 2000). Inappropriate persistence of inflammation leads to tissue damage, which in turn causes further inflammation and more tissue damage. This may be one of the causes of “ age‐related diseases” (Baylis et al. 2013). Some extant findings suggest that efferocytosis is impaired during aging (Aprahamian et al. 2008; Schloesser et al. 2023), which may be closely related to inflammaging. Therefore, the main mechanisms of diminished efferocytosis during aging are discussed below (Figure 3).

#### 3.2.1 Senescent Cells Inhibit Efferocytosis

In aging and various chronic diseases (Coppé et al. 2010) (including pulmonary fibrosis, liver fibrosis, and obesity; Childs et al. 2017), senescent cells gradually accumulate (López‐Otín et al. 2013) and have a negative effect on the body. On the one hand, the senescence‐associated secretory phenotype (SASP) secreted by senescent cells are pro‐inflammatory, which can contribute to the persistence of local inflammation (Coppé et al. 2010). On the other hand, a small proportion of senescent cells can undergo oncogenic mutations that increase the incidence of age‐related malignancies (Sharpless and Sherr 2015).

So why do senescent cells accumulate in the body without being quickly removed? It has recently been shown that senescent cells are resistant to macrophage‐mediated phagocytosis and clearance (Rothlin and Ghosh 2023; Schloesser et al. 2023). Meanwhile, senescent cells prevent macrophages from removing surrounding apoptotic cell bodies through efferocytosis (Schloesser et al. 2023). This effect in senescent cells has been termed senescent cell‐mediated efferocytosis suppression (SCES), and its main mechanism is an increase in the CD47‐modifying enzyme QPCT/L and enhanced CD47 expression in senescent cells (Schloesser et al. 2023). Senescent cells are resistant to apoptosis (Childs et al. 2014). Unlike apoptotic cells, senescent cells express more “don't eat‐me” signals (Gruenwald et al. 2024; Schloesser et al. 2023). CD47 is a “don't‐eat‐me” signal in efferocytosis, which inhibits efferocytosis by binding to SIRPα on the macrophage surface (Logtenberg, Scheeren, and Schumacher 2020). However, SCES can be prevented by blocking the SIRPα–CD47–SHP‐1 axis (Schloesser et al. 2023). In addition, the expression of CD24, a newly identified candidate protein for “don't‐eat‐me” signaling, which interacts with the ITIM‐containing inhibitory receptor Siglec‐10 on macrophages (Barkal et al. 2019), is increased in senescent cells (Schloesser et al. 2023). Most of the “eat‐me” signals are exposed during apoptosis, but senescent cells are resistant to apoptosis (Tower 2015) and have difficulty with spontaneous apoptosis. This may be another reason why senescent cells are not easily removed by phagocytes. However, it is worth noting that some rapidly senescent cells such as erythrocytes and neutrophils express “eat‐me” signals and are cleared by phagocytes (Cockram et al. 2021).

#### 3.2.2 Effects of Aging on Macrophages

First, the internal microenvironment in which macrophages reside is altered during the aging process, affecting the role of macrophages in efferocytosis. In a model of UV radiation–induced keratinocyte apoptosis, removal of apoptotic cells was decreased in older mice (2 years old) compared to that in younger mice (8 weeks) (Aprahamian et al. 2008). Also, in in vitro experiments, when macrophages were pretreated with serum from older mice, their capacity to phagocytose apoptotic particles was diminished in contrast to when macrophages were treated with serum from younger animals (Aprahamian et al. 2008). The phenomenon that in vivo environmental changes under aging conditions may affect macrophage phagocytosis has been reflected in other studies. For example, older mice have more B cells in the peritoneum and more B cell‐derived IL‐10 than younger mice, which impairs phagocytosis by peritoneal macrophages (Linehan et al. 2014).

In the smelling phase of efferocytosis, the ability of macrophages to move and migrate is critical. In an earlier study, it was reported that immune cells from older subjects had a reduced ability to migrate toward chemokines compared to immune cells from younger subjects (Fietta et al. 1994). The ability of macrophages to move also changes with age. For example, the motility of microglia (i.e., brain macrophages) varies with age, with young microglia becoming more motile and readily stretching their branches in the presence of ATP, whereas older microglia are considerably less motile (Damani et al. 2011).

Furthermore, under aging conditions, the efferocytosis‐associated receptors on the surface of macrophages are altered, resulting in an impaired eating phase of efferocytosis. It has been found in previous studies that during inflammation or oxidative stress, the MerTK receptor on the surface of macrophages is cleaved and the extracellular portion of the receptor is shed, resulting in decreased MerTK function (Thorp et al. 2011). Interestingly, senescent cells can secrete large amounts of pro‐inflammatory factors and proteolytic enzymes through the SASP (Campisi and d'Adda di Fagagna 2007). Nicholas Rymut et al. found increased MerTK cleavage in macrophages treated with SASP compared to that in controls, resulting in a significant decrease in efferocytosis (Rymut et al. 2020). Therefore, the increase of SASP during senescence leads to increased MerTK cleavage on the surface of macrophages and consequently efferocytosis defects. At the same time, this experiment also demonstrated that the application of Resolvin D1 can limit senescent cell‐mediated MerTK cleavage, and to a certain extent, it can treat senescence‐induced impaired efferocytosis (Rymut et al. 2020). In another study, the expression of Axl, growth arrest‐specific protein 6 (Gas6), and MerTK, as well as the expression of the efferocytic response gene Abca1, was downregulated in bone marrow macrophages collected from aged mice compared with those from young mice (Frisch et al. 2019). This may be an important reason for the decline in macrophage efferocytosis in aging. Moreover, in human experiments, a decreased ability of macrophages to phagocytose apoptotic polymorphonuclear neutrophils (PMNs) was observed in the elderly by comparing older and younger people (De Maeyer et al. 2020). This is due to a decline in macrophage surface expression of TIM‐4 as a result of increased macrophage p38 MAPK activity in older people. TIM‐4 is a key receptor for macrophage binding to PS. Administration of an oral inhibitor of p38 restores efferocytosis and inflammatory resolution.

A further aspect of interest is that in numerous tissues, the quantity of dying cells greatly surpasses the quantity of macrophages, so that it is often the case that a single macrophage needs to uptake more than one dying cell in succession, a process that is regulated by a variety of mechanisms. One important regulatory mechanism is that mitochondria within macrophages undergo fission mediated by dynamin‐related protein 1 (Drp1) (Wang et al. 2017), which promotes calcium release from the endoplasmic reticulum to drive phagolysosome–lysosome fusion and vesicular transport, thereby enabling macrophages to undergo continuous efferocytosis. However, reduced activity of Drp1, which is involved in mitochondrial fission, and dysregulation of mitochondrial fission were observed during senescence (Sharma et al. 2019). Thus, the capacity of macrophages for continuous efferocytosis may be reduced by dysregulated mitochondrial fission.

What is more, another reason for the diminished efferocytosis of macrophages as a result of aging may be the transcription factor, Krüppel‐like factor 4 (Klf4), with a role in regulating cell differentiation and reprogramming, whose expression is selectively reduced in aging macrophages (Blacher et al. 2022). Related studies have shown that knockdown of Klf4 in macrophages significantly disrupts phagocytosis. In young macrophages, the diurnal increase in Klf4 gene expression resembled the diurnal peak of phagocytosis. In aged macrophages, the diurnal peak of Klf4 gene expression decreased, as did the diurnal peak of phagocytosis. Thus, Klf4 is involved in altered phagocytosis in aged macrophages (Blacher et al. 2022; Frisch et al. 2019). However, it is worth noting that some studies have observed that phagocytosis of microglia and bone marrow macrophages/monocytes is not impaired by aging (Linehan et al. 2014; Yanguas‐Casás et al. 2020).

In addition, the phenotype of macrophages changes with age. In a study comparing 18‐ to 20‐month‐old mice with 10‐ to 12‐week‐old young mice, mRNA expression of the M2‐related genes was lower in splenic macrophages from older mice compared with macrophages from young mice pre‐stimulated with IL‐4 (Mahbub, Deburghgraeve, and Kovacs 2012). However, in vitro‐differentiated bone marrow‐derived macrophages did not exhibit age‐related polarization impairment.

Also, it is worth mentioning that senescent cells accumulated during aging recruit macrophages and can induce macrophage senescence (Behmoaras and Gil 2021), whereas senescent macrophages typically exhibit an imbalanced polarization state, impaired phagocytosis and impaired migration (Gu et al. 2024). This could be another potential reason for the decline in macrophage efferocytosis during aging.

In summary, during aging, multiple factors such as changes in the in vivo microenvironment, decreased macrophage migration and motility, altered efferocytosis‐associated receptors on the macrophage surface, and mitochondrial dysfunction cooperate to cause efferocytosis defects.

#### 3.2.3 Effects of Aging on Neutrophils

Neutrophil chemotaxis and migration are the basis for their physiological functions. Existing studies have demonstrated that aging affects neutrophil chemotaxis and migration (Niwa et al. 1989; Sapey et al. 2014; Wenisch et al. 2000). In aged mice, both the speed and direction of neutrophil movement were found to become sluggish (Nomellini et al. 2012; Ren et al. 2009; Toapanta and Ross 2009). Chen et al. showed that neutrophils migrated incorrectly in a lung model of *Pseudomonas aeruginosa* infection. In infected older mice, neutrophils were localized in the lung parenchyma, whereas in infected younger mice neutrophils observed were located in the alveolar space (Chen et al. 2014). Similar results were observed in a mouse model of lung inflammation due to thermal injury (Nomellini et al. 2012). Similar to the results of the mice experiments, in human in vitro experiments, neutrophils isolated from the elderly exhibit reduced chemotaxis. Neutrophil chemotaxis to stimuli such as FMLP and GMCSF decreases significantly with age (Fulop et al. 2004). Similarly, Sapey et al. have demonstrated that neutrophils migrate inaccurately in response to stimuli in the elderly compared to that in the young (Sapey et al. 2014). This behavior of neutrophils is due to constitutive activation of the PI3K pathway, and the inhibition of this pathway restores migratory accuracy (Sapey et al. 2014). In aging, neutrophil migration is impaired, which affects their response rate to inflammation as well as their efferocytosis function.

## 4 Efferocytosis Promotes Tumor Growth and Metastasis by Inhibiting Inflammatory Responses and Antitumor Immunity

**FIGURE 4:**
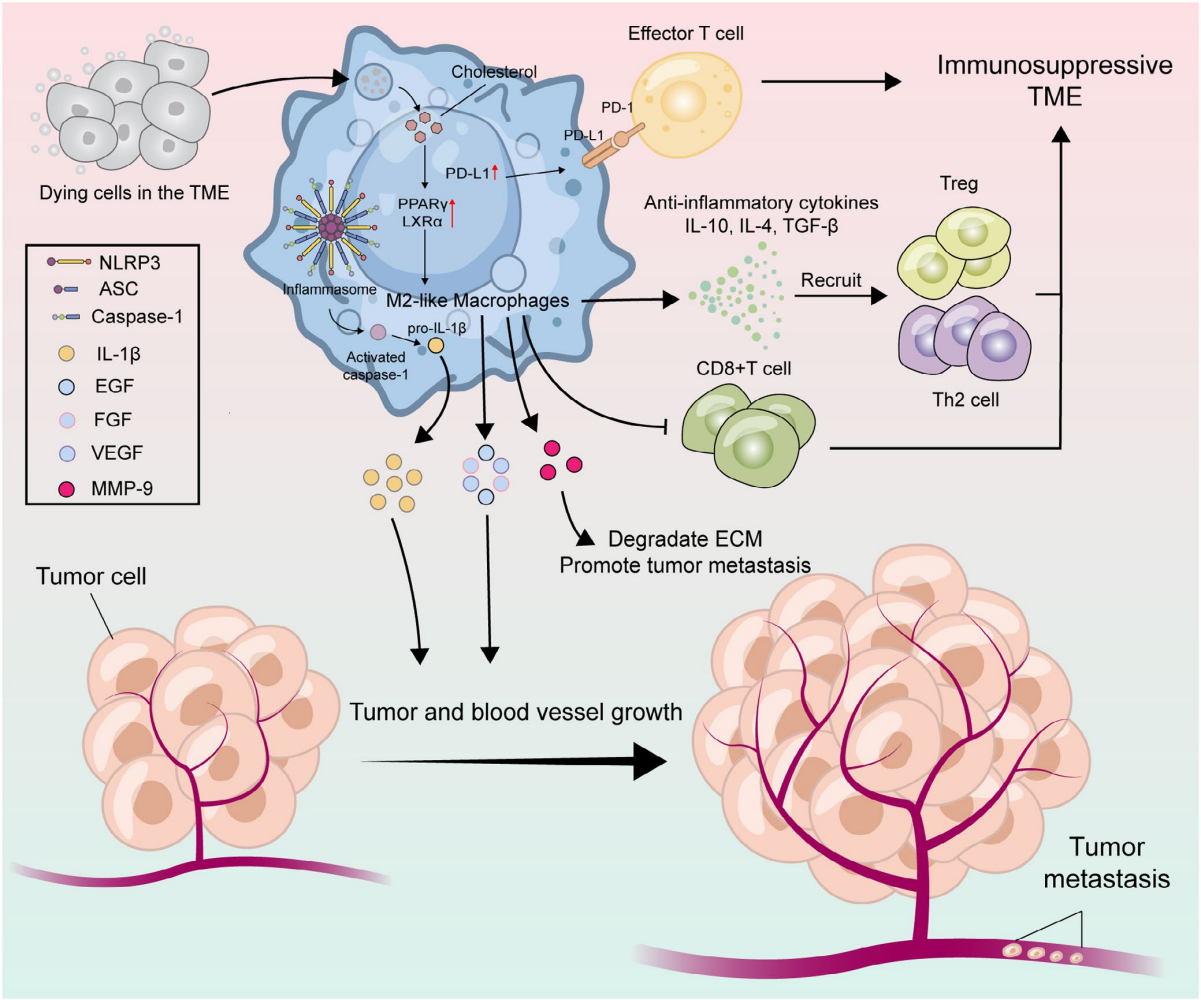
Role of efferocytosis in tumor. Efferocytosis promotes the formation of immunosuppressive TME, tumor growth, and metastasis. First, after phagocytosis of dying cells in the TME by TAMs, cholesterol, a product of digestion, activates PPAR‐γ and LXR‐α, which subsequently, through a downstream signaling pathway, polarizes the TAMs toward the M2 phenotype. M2‐like macrophages secrete anti‐inflammatory cytokines, for example, IL‐4, IL‐10, and TGF‐β, which recruit Treg, and Th2 cells. These anti‐inflammatory factors recruit Treg, Th2 cells, which participate in the formation of an immunosuppressive TME. Reduced antigen presentation to T cells by M2‐like macrophages suppresses CD8^+^ T cells, ultimately impairing antigen‐dependent antitumor immunity. In addition, the efferocytosis process induces upregulation of PD‐L1. PD‐L1 binds to PD‐1 receptor expressed on tumor–infiltrating activated T cells, leading to negative regulation of tumor‐reactive T cell activation and attenuation of antitumor T cell responses. Efferocytosis directly activates the NLRP3 inflammasome in macrophages, which activate downstream caspase‐1 and lead to subsequent IL‐1β secretion, thereby promoting tumor growth. M2‐like macrophages produce growth factors, such as EGF, FGF, and VEGF, which promote tumor vascularization. Efferocytosis directly activates nlrp3 inflammatory vesicles in macrophages, which activate downstream caspase‐1 and lead to subsequent IL‐1β secretion, thereby promoting tumor growth. M2‐like macrophages produce growth factors, such as EGF, FGF, and VEGF, which promote tumor vascularization. These new blood vessels provide the main pathway for tumor cells to leave the primary tumor site and enter the circulation, thus promoting metastatic spread of the tumor. Finally, M2‐like macrophages also secrete MMP‐9, which digests the extracellular matrix and promotes tumor metastasis.

The anti‐inflammatory properties of efferocytosis have a positive effect in the fight against inflammatory diseases. However, this anti‐inflammatory property in tumors suppresses antitumor immunity, which in turn promotes tumor growth and has a negative impact on human health (Figure 4).

### 4.1 Formation of a Tumor‐Tolerant and Immunosuppressive TME

After efferocytosis of apoptotic tumor cells by macrophages, they are converted to an immunosuppressive M2 phenotype (Allavena et al. 2008) through metabolic signaling downstream of PPAR‐γ and LXR‐α (Werfel and Cook 2018) (Figure 4).

Efferocytosis upregulates the expression and activity of MerTK, Axl, and Tyro3 on macrophages, facilitating differentiation to an immunosuppressive phenotype. We already know that macrophages are important antigen‐presenting cells that serve as vital for both innate and adaptive immunity. Thus, efferocytosis‐mediated shifts in macrophage phenotypes result in reduced cross‐presentation of antigen to T cells, which in turn leads to reduced T cell clonal expansion and ultimately to damaged antigen‐dependent antitumor immunity (Tajbakhsh, Farahani, et al. 2021; Tajbakhsh, Gheibi Hayat, et al. 2021; Werfel and Cook 2018). Nguyen et al. showed that Mertk on the surface of macrophages induced upregulation of programmed cell death 1 ligand 1 (PD‐L1) transcription in apoptotic cells (Nguyen et al. 2014). PD‐L1 binds to PD‐1 expressed on tumor‐infiltrating activated T cells, leading to negative regulation of tumor‐reactive T cell activation and attenuation of antitumor T cell responses (Dong et al. 2002; Freeman et al. 2000; Iwai et al. 2002). Later, other researchers found that PS enhanced PD‐L1 signaling and that the PS‐TAM receptor–PD‐L1–PI3K/ Akt signaling axis may promote tumor immune escape and chemotherapy resistance (Kasikara et al. 2017).

### 4.2 Efferocytosis Promotes Tumor Growth

Efferocytosis directly activates signaling of NLRP3 inflammasome, thereby driving IL‐1β secretion, which promotes tumor growth (Lang et al. 2022). Several research groups have demonstrated that one key way to encourage tumor growth in vivo is the activation of myeloid intrinsic inflammasomes (Lang et al. 2022; Zeng et al. 2018). The activation of NLRP3‐dependent inflammasome signaling in macrophages is directly triggered by macrophage‐mediated efferocytosis in TME, which activates downstream caspase‐1 and leads to subsequent secretion of IL‐1β (Lang et al. 2022). IL‐1β secreted by macrophages can promote tumor growth (Lang et al. 2022; Ridker et al. 2017). In summary, during efferocytosis, downstream effects of NLRP3/Caspase‐1/IL‐1β signaling axis in macrophages promote tumor growth in vivo.

Furthermore, overexpression of a variety of efferocytosis‐related ligands and receptors has been found to contribute to tumor cell growth and tumorigenesis (Rothlin et al. 2007). The TAM receptor family, including MerTK, Axl, Tyro3, is the important molecule involved in efferocytosis processes (Rothlin et al. 2007; Verma et al. 2011). Activating Axl via PI3K/Akt and MAPK/Erk signaling pathways can contribute to the survival and rapid growth of tumor cells (Rothlin et al. 2007; Tajbakhsh, Farahani, et al. 2021; Tajbakhsh, Gheibi Hayat, et al. 2021).

### 4.3 Efferocytosis Promotes Tumor Metastasis

Efferocytosis not only induces the formation of an immunosuppressive TME and promotes tumor growth but also contributes to tumor metastasis. We already know that efferocytosis induces macrophage polarization toward the M2 phenotype. It has been shown that M2 macrophages produce growth factors such as epidermal growth factor (EGF), fibroblast growth factor (FGF), and vascular endothelial growth factor (VEGF) (Asare et al. 2020; Lewis et al. 2000), which in tumors promote the growth of tumor blood vessels, so that the tumor receives an adequate blood supply. In parallel, these neovessels provide the main pathway for tumor cells to enter the circulation from the site of the primary tumor, thus enhancing metastatic spread of the tumor.

In addition, secretion of matrix metallopeptidase‐9 (MMP‐9) by M2 macrophages contributes to the degradation of extracellular matrix (ECM), which encourages cancer cells to invade and disseminate to far‐off locations in vivo (Vinnakota et al. 2017). This phenomenon has been demonstrated in both oral squamous cell carcinoma and colorectal cancer (Chiu et al. 2015; Vinnakota et al. 2017). What is more, Chen et al. have demonstrated that chitinase 3‐like protein 1 (CHI3L1) secreted by tumor‐infiltrating M2 macrophages promotes the metastasis of gastric and breast cancer cells in vitro and in vivo (Chen et al. 2017). Moreover, it is noteworthy that a highly metastatic melanoma cell line, B16F10, was found to produce a large number of PS‐containing microvesicles in vitro. These microvesicles induced melanoma metastasis in BALB/c mice (Lima et al. 2009).

In studies related to breast cancer, the pro‐tumoral and metastatic characteristics of breast malignancies in parous women have been linked to significant cell death and efferocytosis (Stanford et al. 2014). Enhanced efferocytosis during postpartum involution induces macrophages to polarize toward M2 and secrete wound‐healing cytokines, thereby promoting tumor progression and metastasis.

### 4.4 Another Possible Side of Efferocytosis: Antitumor Effect of Inhibiting Inflammatory Responses

As mentioned earlier, most current academic studies believe that the role of efferocytosis in tumors is to promote tumorigenesis, development, and metastasis, which can be detrimental to the treatment and recovery of tumor patients. However, there is also evidence that specific inflammatory conditions can influence tumor promotion (Mantovani et al. 2008). A team of researchers found that macrophages expressing the TAM receptors Axl and Mertk phagocytose apoptotic neutrophils by efferocytosis and reduce the secretion of pro‐inflammatory factors, thereby suppressing long‐term chronic pro‐tumor inflammation and reducing the incidence of inflammation‐associated colorectal cancer (Bosurgi et al. 2013).

Additionally, the Gas6/TAM inhibitory mechanism in intestinal cancers has been steadily identified. Gas6 is a humoral ligand for the TAM receptor (van der Meer, van der Poll, and van 't Veer 2014). Gas6 acts as a bridging protein for macrophages binding to dead cells during efferocytosis. Interestingly, a study demonstrated that Gas6 deficiency enhances colitis‐associated tumorigenesis in mice (Akitake‐Kawano et al. 2013). Gas6 may decrease the immunological response of colonic mesenchymal cells, which could explain its suppressive action on intestinal cancers. Localized increases in Gas6 trigger the TLR/Gas6/TAM signaling cascade, restricting the release of pro‐inflammatory molecules and NF‐κB activation. Consequently, pro‐tumor proteins Cox2 and c‐Myc were also downregulated, along with the activation of suppressor of cytokine signaling 1/3 (SOCS1/3), which limits intestinal inflammation by inhibiting immune responses from stromal monocyte lineage cells (Lemke and Rothlin 2008).

In summary, the efferocytosis‐related signaling described above has been shown to reduce local immune‐inflammatory responses, exerting a potential inhibitory effect on inflammation‐associated intestinal tumors. Although the current study is limited to colorectal inflammation‐related tumors, we believe that efferocytosis may have some similar inhibitory effect on other inflammatory precancerous lesions or inflammatory precancerous diseases to inhibit their progression to cancer.

## 5 Efferocytosis Regulations by Tumor Microenvironment Factors

In the previous sections, we have elaborated that phagocyte removal of dead cells by efferocytosis is an immunosuppressive phenomenon in the tumor microenvironment, which counteracts antitumor immunity and promotes tumor growth and metastasis. Efferocytosis is regulated in the TME by a variety of factors, such as cytokines, cellular metabolites, and alterations in the microenvironment (Table 2). Currently, relevant studies have focused on the effects of tumor microenvironmental factors on phagocytosis and PS externalization during efferocytosis. The next sections discuss the mechanisms and effects of various factors in the tumor microenvironment that regulate efferocytosis, mainly from these two aspects.

**TABLE 2:** Molecules regulating efferocytosis in the tumor microenvironment.

Molecule	Effect on efferocytosis	Mechanisms regulating efferocytosis	Ref.
C1q	Positive	C1q promotes efferocytosis by promoting macrophage polarization toward the M2 phenotype	Galvan et al. (2012)
Adiponectin	Positive	Adiponectin induces the expression of MerTK in macrophages and promotes efferocytosis through an AMPK‐mediated signaling pathway	Galvan et al. (2014)
CRT	Both positive and negative effects	CRT on the surface of apoptotic cells binds to C1q to promote efferocytosis, but CRT released extracellularly inhibits efferocytosis after uptake by macrophages	Osman et al. (2017)
NRF1	Negative	Under hypoxic conditions, inhibition of NRF1 degradation impairs the polarization of tumor‐associated macrophages, thereby inhibiting efferocytosis	Ma et al. (2019)
Putrescine	Positive	Putrescine increases the activity of the small GTPase Rac1, which promotes actin rearrangement and subsequent efferocytosis	Yurdagul et al. (2020)
ROS	Positive	ROS induces PS exposure by oxidizing membrane lipids and generating calcium influxes that activate PS exporters	Choo et al. (2012)
COX2	Positive	COX2 enhances efferocytosis by promoting macrophage binding to apoptotic cells	Meriwether et al. (2022)
WDFY3	Positive	WDFY3 protein promotes LC3 lipidation and subsequent lysosomal acidification	Shi et al. (2022)
TRPM7	Positive	TRPM7 mediates a pH‐activated cationic current necessary to sustain phagosomal acidification	Schappe et al. (2022)

Abbreviations: COX2, cyclooxygenase 2; CRT, calreticulin; NRF1, nuclear respiratory factor 1; ROS, reactive oxygen species.

### 5.1 Effects of TME on Phagocytes

#### 5.1.1 Complement and Adiponectin

Complement proteins are present in the TME, and their activation can be detected in the progression of a range of tumors (Afshar‐Kharghan 2017). Exposed PS on the outer surface of the plasma membrane of apoptotic cells can activate complement, resulting in complement proteins that easily cover the surface of apoptotic cells. C1q binds to apoptotic cells and acts as a bridge between apoptotic cells and phagocytes via CRT and CD91. Complement makes it easier for macrophages to ingest apoptotic cells, thus promoting macrophage efferocytosis (Mevorach et al. 1998). Furthermore, MerTK and Gas6 expression is upregulated in mouse bone marrow‐derived macrophages and peritoneal macrophages stimulated by C1q (Galvan et al. 2012). Adiponectin is known as adipokine, which is produced by adipocyte and released into the circulation (Kadowaki and Yamauchi 2005). Adiponectin signaling can activate adenosine monophosphate‐activated protein kinase (AMPK) and PPAR‐α (Heiker, Kosel, and Beck‐Sickinger 2010). And stimulation of macrophages with C1q also leads to activation of AMPK. Adiponectin and C1q elicit MerTK expression and promote MerTK‐dependent efferocytosis through AMPK‐mediated signaling pathway in macrophages (Galvan et al. 2014).

#### 5.1.2 Calreticulin

Calreticulin (CRT) is a multifunctional protein found primarily in the lumen of the endoplasmic reticulum (Schcolnik‐Cabrera et al. 2019). After translocation of CRT to the plasma membrane, it acts as an ‘eat‐me’ signal to enhance efferocytosis of ACs and clearance of tumor cells (Kielbik, Szulc‐Kielbik, and Klink 2021; Wijeyesakere et al. 2016). In the early stages of apoptosis, CRT is released in large quantities into the surrounding environment and is endocytosed by macrophages, leading to an acceleration of macrophage migration, an increase in the secretion of pro‐inflammatory chemokines (IL‐8), and a decrease in the phagocytosis of apoptotic cells (Osman et al. 2017).

#### 5.1.3 Nuclear Respiratory Factor 1 (NRF1)

A recent study has shown that the SIAH2–nuclear respiratory factor 1 (NRF1) axis reshapes the TME by modulating several processes such as tumor mitochondrial function, TAM polarization, and cell death (Ma et al. 2019). When the tumor microenvironment is in hypoxia, the hypoxia‐induced E3 ligase SIAH2 degrades NRF1, which downregulates the expression of nuclear‐encoded mitochondrial genes, leading to an enhanced pro‐tumor immune response.

#### 5.1.4 Putrescine

Macrophages use the arginine and ornithine extracted from the previously engulfed apoptotic cells to facilitate subsequent efferocytosis (Yurdagul et al. 2020). Arginine and ornithine can be digested to putrescine by arginase and ornithine decarboxylase expressed by macrophages. Putrescine may raise the activity of the small GTPase Rac1, which in turn promotes actin rearrangement and efferocytosis.

### 5.2 Effects of TME on PS Externalization

Under normal conditions, PS is largely absent from the outer surface of mammalian cell plasma membranes. PS is exposed to the cell surface during apoptosis, necrosis, cell injury, cell activation, and malignant transformation (Ran, Downes, and Thorpe 2002). Increased externalization of PS as an “eat‐me” signal facilitates phagocytosis efferocytosis. So what are the factors that lead to PS externalization of tumor cells in the TME?

Sophia et al. showed that PS is exposed to the luminal surface of various tumor vascular endothelial cells and demonstrated that in vitro hypoxia/re‐oxidation leads to a significant increase in PS exposure in endothelial cells (Ran and Thorpe 2002). This effect can be amplified by other stimuli such as inflammatory factors (IL‐1 or TNFα), acidity, and thrombin. The mechanisms by which the above factors lead to PS externalization may be, on the one hand, hypoxia/reoxygenation and thrombin can generate ROS in endothelial cells through the activation of NADPH oxidase‐like membrane enzyme (Zulueta et al. 1995). ROS production by malignant cells leads to endothelial cell damage (Shaughnessy et al. 1989), which in turn stimulates PS externalization. On the other hand, tumor cells and the vascular system experience metabolic stress as a result of the hostile tumor microenvironment, which raises intracellular calcium and activates calcium‐activated PS scramblases such as TMEM16F and its associated members (Gyobu et al. 2017).

Furthermore, both radiotherapy and chemotherapy lead to increased PS exposure of tumor cells (He et al. 2009), which is probably caused by ROS generated by the chemotherapeutic drugs and irradiation (Leach et al. 2001). It is believed that ROS either oxidize membrane lipids and cause calcium influxes that activate PS exporters (Bitbol et al. 1987) or they activate acid sphingomyelinase A to produce ceramide, which directly enhances PS transbilayer membrane movement (Kolesnick and Fuks 2003).

Finally, an important source of PS in the TME comes from PS‐positive microvesicles (Lima et al. 2009) and PS‐positive exosomes (Lea et al. 2017), which contribute to the surface area of externalized PS. According to a study by Fujii et al., TMEM16F activated by elevated intracellular Ca^2+^ supported not only PS externalization but also the release of PS‐positive particles (Fujii et al. 2015).

### 5.3 Other Regulatory Factors of Efferocytosis in TME

According to a recent study, COX2 was demonstrated to be one of the factors located inside macrophages that regulate efferocytosis (Meriwether et al. 2022). COX2 enhances efferocytosis by promoting macrophage binding to apoptotic cells. In addition, Shi et al. (2022) through a genome‐wide CRISPR knockdown screen of primary mouse macrophages found that if WDFY3 is knocked out of macrophages, it leads to impaired actin disassembly, thereby specifically impairing macrophage uptake of apoptotic cells (Shi et al. 2022). Meanwhile, WDFY3 protein is involved in regulating LAP and degradation of apoptotic cells by promoting LC3 lipidation and subsequent lysosomal acidification. Therefore, it is reasonable to assume that WDFY3 is a factor in macrophages that regulates efferocytosis. Additionally, new advances have been made in the study of factors regulating phagosome maturation during efferocytosis. TRPM7 has been shown to be a central regulator of phagosome maturation (Schappe et al. 2022).

## 6 Therapeutic Potential of Efferocytosis in Tumors and Age‐Related Diseases

### 6.1 Therapeutic Application of Efferocytosis in Tumors

In cancerous tissues, the anti‐inflammatory and immunosuppressive effects produced by efferocytosis promote cancer progression. In addition, conventional treatments for cancer (e.g., chemotherapy and radiotherapy) have resulted in increased apoptosis of cancer cells, which exacerbates the effects of efferocytosis, as well as the amplification of the immunosuppressive effects produced by efferocytosis. Hence, the treatment of targeted efferocytosis process as an adjuvant therapy can be combined with traditional anticancer therapies to improve the effectiveness of cancer treatment and improve the prognosis of cancer patients. Next, we summarize the targeted therapies for the different stages of efferocytosis.

#### 6.1.1 Blocking the Eating Process

In recent years, the research on PS‐targeted drugs has been gradually deepened, and PS‐targeted drugs mainly include PS‐targeted antibodies and membrane‐linked proteins (Table 3). Annexin proteins are naturally occurring PS ligands in vivo that preferentially bind to PS with high affinity and inhibit macrophage uptake of apoptotic cells (Munoz et al. 2007). Blockade of efferocytosis by annexin proteins triggers a pro‐inflammatory response (Munoz et al. 2007) that enhances antitumor immunity. Systemic administration of annexin proteins can slow tumor progression (Lima et al. 2009). PS‐targeting antibodies are synthetic drugs that bind specifically to PS with high affinity. PS is not only expressed on the surface of apoptotic tumor cells but also exposed on the luminal surface of the tumor vascular endothelium (He et al. 2009). Consequently, antiphosphatidylserine antibody not only increases antitumor immunity by affecting efferocytosis but also leads to tumor vascular occlusion (DeRose, Thorpe, and Gerber 2011). Preclinical oncology studies have shown that PS's targeting antibodies 3G4, 2aG4, and mch1N11 produce potent antitumor effects when combined with chemotherapy or radiotherapy (Birge et al. 2016).

**TABLE 3:** Clinical trials of PS‐targeted therapy.

Treatment	Tumor type	Clinical trial phase	Status	Identifier
Bavituximab	Advanced solid tumor	Phase 1	Completed	NCT00129337
Bavituximab with radiation and temozolomide	Newly diagnosed glioblastoma	Phase 2	Completed	NCT03139916
Bavituximab and sorafenib plus SBRT	Unresectable hepatocellular carcinoma	Phase 1	Withdraw	NCT02989870
Bavituximab and pembrolizumab	Advanced gastric and GEJ cancer	Phase 2	Completed	NCT04099641
	Advanced hepatocellular carcinoma	Phase 2	Active, not recruiting	NCT03519997
	Squamous cell carcinoma of head and neck	Phase 2	Active, not recruiting	NCT04150900
Bavituximab and sorafenib	Advanced liver cancer	Phase 1/2	Completed	NCT01264705
Bavituximab and paclitaxel	Her2‐negative metastatic breast cancer	Phase 1	Completed	NCT01288261
Bavituximab in combination with capecitabine and radiation	Rectal adenocarcinoma	Phase 1	Completed	NCT01634685
Bavituximab plus docetaxel	Advanced breast cancer	Phase 2	Completed	NCT00669591
	Late‐stage non‐squamous NSCLC	Phase 3	Completed	NCT01999673
Bavituximab plus paclitaxel and carboplatin	Carcinoma breast stage IV	Phase 2	Completed	NCT00669565
	NSCLC	Phase 2	Completed	NCT00687817
Bavituximab and gemcitabine	Previously untreated stage IV pancreatic cancer	Phase 2	Completed	NCT01272791
Bavituximab plus carboplatin and pemetrexed	Chemotherapy‐naive stage IV nonsquamous NSCLC	Phase 1	Completed	NCT01323062

Abbreviations: GEJ, gastroesophageal; NSCLC, non‐small cell lung cancer; SBRT, stereotactic body radiation therapy.

Second, drugs targeting the TAM receptor are being developed, including MerTK inhibitors and Axl inhibitors, which may have the potential to block efferocytosis in the tumor microenvironment (Table 4). A team has been recently developed that nano‐BMS (a nano‐MerTK inhibitor encapsulating BMS777607) is able to inhibit efferocytosis (Wu et al. 2023). Following intratumoral injection of nano‐BMS in mice, apoptotic cells that are not cleared by efferocytosis will undergo immunogenic secondary necrosis and macrophages will be polarized to a pro‐inflammatory M1 phenotype. The anticancer drugs targeting Axl have been described in detail in other reviews (Zhu, Wei, and Wei 2019), and we will not repeat them here.

**TABLE 4:** Summary of clinical trials targeting TAM receptor.

Drug names	Target(s)	Condition(s)	Clinical trial phase	Status	Identifier
Enapotamab vedotin/HuMax‐AXL‐ADC	AXL	Ovarian cancer, cervical cancer, endometrial cancer, NSCLC, thyroid cancer, melanoma, sarcoma, solid tumors	Phase 1/2	Completed	NCT02988817
RXDX‐106	TYRO3, AXL, and MER	Advanced or metastatic solid tumors	Early Phase 1	Terminated	NCT03454243
CCT301‐38	AXL	Relapsed or refractory AXL‐positive sarcomas	Phase 1	Recruiting	NCT05128786
BA3011/CAB‐AXL‐ADC	AXL	NSCLC	Phase 2	Recruiting	NCT04681131
		Undifferentiated pleomorphic sarcoma, liposarcoma, synovial sarcoma, osteosarcoma, Ewing sarcoma	Phase 1/2	Recruiting	NCT03425279
BGB324	AXL	Brain and central nervous system tumors	Early Phase 1	Suspended	NCT03965494
		Metastatic lung cancer, NSCLC Stage IV, adenocarcinoma of lung	Phase 2	Completed	NCT03184571
		Triple negative breast cancer, inflammatory breast cancer Stage IV	Phase 2	Terminated	NCT03184558
		Non‐small cell lung carcinoma	Phase 1	Active, not recruiting	NCT02922777
		Melanoma	Phase 1/2	Active, not recruiting	NCT02872259
Q702	AXL, MER, CSF1R	Solid tumor, advanced cancer, metastatic cancer	Phase 1	Recruiting	NCT04648254
AVB‐S6‐500	AXL	Urothelial carcinoma	Phase 1	Active, not recruiting	NCT04004442
Bemcentinib	AXL	Cancer of pancreas	Phase 1/2	Terminated	NCT03649321
TP‐0903	AXL	Advanced solid tumors	Phase 1	Completed	NCT02729298
SLC‐391	AXL	Solid tumor	Phase 1	Completed	NCT03990454
XZB‐0004	AXL	Advanced solid tumor, NSCLC	Phase 1	Not yet recruiting	NCT05772455
MRX‐2843	MER	Advanced NSCLC	Phase 1	Recruiting	NCT04762199

In addition, MFG‐E8 is highly expressed in a variety of cancers (Geoffroy et al. 2022). It reprograms macrophages to a pro‐tumor/pro‐angiogenic M2 phenotype (Brissette et al. 2012), which is commonly associated with tumor growth and poor patient prognosis (Jia et al. 2017). It has been demonstrated that the combination of MFG‐E8‐targeted therapy and chemotherapy significantly improves antimelanoma treatment outcomes compared to chemotherapy alone (Zhao et al. 2015). An alkaloid derived from the yellow dock (i.e., coptisine) was found to inhibit tumor growth and progression by downregulating MFG‐E8 in colorectal cancer (Cao et al. 2018).

#### 6.1.2 Blocking LAP

During efferocytosis, LAP is an indispensable part of efferocytosis process (Martinez et al. 2011). According to previous studies, Rubicon is a central component of the LAP pathway (Wong, Sil, and Martinez 2018). In preclinical studies, inhibition of Rubicon limits tumor growth and enhances immune activation by reducing LAP‐mediated efferocytosis. Furthermore, vacuolar‐type H (+)‐ATPase (V‐ATPase) activity was recently shown to be required for LAP (Florey et al. 2015), and inhibition of V‐ATPase prevents LAP by blocking LC3 lipidation. This provides a potential new therapeutic target for regulating LAP.

#### 6.1.3 Reversing Macrophage M2 Polarization

Macrophage polarization toward the M2 phenotype during efferocytosis is an important contributor to the immunosuppressive TME. According to a recent study, thymosin α‐1 (Tα‐1) significantly reverses M2 polarization of TAM during efferocytosis in the breast tumor microenvironment (Wei et al. 2022). In an in vivo breast cancer model, Tα‐1 in conjunction with Epirubicin treatment markedly slowed down the growth of the tumor by decreasing IL10 produced by macrophages and increasing the quantity and quality of CD4^+^ and CD8^+^ T cells that infiltrate the tumor.

### 6.2 Therapeutic Application of Efferocytosis in Age‐Related Diseases

Several age‐related diseases, such as atherosclerosis, hypertension, diabetes mellitus, and osteoarthritis, have been demonstrated to be associated with impaired efferocytosis (Boada‐Romero et al. 2020; Doran et al. 2017; Doran, Yurdagul, and Tabas 2019; Xiong et al. 2024; Zhang, Liu, et al. 2024; Zhang, Wei, et al. 2024). Conventional therapies combined with enhanced efferocytosis may improve the outcome of these age‐related diseases.

As mentioned previously, MerTK cleavage during aging reduces MerTK in macrophages in aged mice, which can lead to impaired efferocytosis. It has been shown that Resolvin D1 can prevent MerTk cleavage during aging to improve efferocytosis (Rymut et al. 2020). Therefore, treatment with Resolvin D1 may be a novel adjunctive therapy for age‐related diseases. In addition, decreased TIM‐4 expression in macrophages in the elderly is associated with altered p38 MAPK activity, which impairs efferocytosis and affects inflammatory regression (De Maeyer et al. 2020). Losmapimod, a p38 inhibitor used in clinical development, restores TIM‐4 expression and efferocytic activity in macrophages generated in vitro in elderly people for the treatment of impaired efferocytosis during the aging process (Zhang, Wei, et al. 2024).

In addition, atherogenesis is associated with upregulation of CD47 (Kojima et al. 2016). CD47 is a “don't eat‐me” signal that makes cells resistant to efferocytosis. Restriction of efferocytosis leads to pathologic accumulation of apoptotic cells in diseased vessels and secondary necrosis (Ngai, Sukka, and Tabas 2024). Inhibition of CD47 with an anti‐CD47 antibody has been shown to reverse this pathology, removing apoptotic debris from atherosclerotic plaques, which may be useful for treating atherosclerosis in the elderly (Kojima et al. 2016). In a recent related study, researchers developed pro‐efferocytic nanoparticles to enhance efferocytosis and inflammation resolution in order to increase the precision of targeted therapy (Patel et al. 2024). The nanoparticles are expected to be a novel drug for atherosclerosis.

Hypertension is recognized as a chronic inflammatory disease (Zhang, Liu, et al. 2024), and promoting the reduction of inflammation is critical to the treatment of hypertension. There is evidence that treatment with gastrin alleviates Ang II‐mediated inhibition of macrophage efferocytosis, which reduces renal inflammation and normalizes blood pressure (Gu et al. 2021). Mechanistically, gastrin, an agonist of cholecystokinin receptor B (CCKBR), enhances PPAR‐α transcription by promoting CCKBR nuclear translocation and promotes efferocytosis of apoptotic renal tubular cells by macrophages, thereby ameliorating renal fibrosis and lowering blood pressure.

Chronic nonhealing wounds are a common complication in diabetic patients, which is associated with impaired efferocytosis in wound tissue (Khanna et al. 2010). It has been found that the membrane transporter SLC7A11 is a brake for efferocytosis, which is highly expressed within the wounds of diabetic mice (Maschalidi et al. 2022). Targeting SLC7A11 accelerates diabetic wound healing by enhancing efferocytosis of dendritic cells. Recent studies have shown that both ginsenoside Rg5 (Xia et al. 2023) and isoliquiritigenin (Gong et al. 2024) can alleviate SLC7A11‐mediated efferocytosis inhibition to promote wounds healing in diabetes.

Osteoarthritis is a prevalent disease of old age, and the key factor in its pathology is chondrocyte senescence. As it has been explained in the previous section that senescent cells cannot be removed by macrophages through efferocytosis and even impede efferocytosis, therefore, apoptosis of senescent chondrocytes at the lesion site can be induced to promote the removal of these chondrocytes by macrophages. Wei Xiong et al. developed the cartilage lesion‐localized hydrogel microspheres with pro‐apoptotic liposomes, which can remodel the in situ efferocytosis and recruit endogenous stem cells to accelerate cartilage repair (Xiong et al. 2024). In vivo experiments have demonstrated that these microspheres reverse cartilage aging and promote cartilage repair in OA patients.

## 7 Conclusion and Perspectives

Phagocytes remove dead cells from the body through efferocytosis, preventing secondary necrosis and inhibiting the inflammatory response. As the body gradually ages, the inhibition of efferocytosis, in addition to SASP production by senescent cells, may be one of the major reasons for the presence of low‐level chronic inflammation in the elderly. Thus, in normal humans, efferocytosis is essential for the maintenance of physiological balance and homeostasis of the internal environment. However, the effects of efferocytosis in inhibiting the inflammatory response and promoting the generation of an immunosuppressive microenvironment are extremely detrimental to tumor patients. This is because these properties of efferocytosis inhibit antitumor immunity, promote tolerance and immunosuppressive TME formation, and contribute to the promotion of tumor growth and metastasis. In particular, this immunosuppressive effect of efferocytosis is amplified after chemotherapy or radiotherapy induces apoptosis in a large number of tumor cells, resulting in suboptimal antitumor therapy. However, let us think about it from another point of view: There are some types of tumors that are caused by long‐term chronic inflammation, so the anti‐inflammatory effect of efferocytosis may have an inhibitory effect on the formation of these types of tumors. This idea has been confirmed by some studies. Therefore, we conclude that while efferocytosis has a promotional effect on tumors that have already developed, efferocytosis has a role in controlling the progression of certain precancerous lesions of an inflammatory nature to become cancer.

For the study of the mechanism of efferocytosis, a large number of studies are based on the efferocytosis of apoptotic cells, whereas the efferocytosis of cells that die through other pathways needs to be further explored. In the clinical application of efferocytosis, targeting efferocytosis can increase tumor immunogenicity and inhibit tumor development and metastasis. At present, some efferocytosis‐targeted drugs have entered clinical trials, and we expect that these drugs will show excellent performance in tumor treatment. In addition, the combination of radiotherapy, chemotherapy, and efferocytosis‐targeted therapy may become an important antitumor therapy in the future. However, it deserves special attention that efferocytosis exists widely in human tissues and organs, and how to accurately target efferocytosis in malignant tumors is still unclear. Whether the receptors and ligands associated with efferocytosis in different tissues are differentiated still requires further study.

In recent times, there has been an influx of research in the area of efferocytosis as applied to age‐related diseases. Combination therapies that induce apoptosis and increase efferocytosis in senescent cells may be a way to extend healthy lifespan and improve the quality of life of the elderly. In the future, we can promote molecular targeting and drug therapy in clinical trials through large‐scale preclinical experiments, mainly using large animal models, especially non‐human primates.

## Author Contributions

Z.Q.L. and Y.Q.R. provided direction and guidance throughout the preparation of this manuscript. Y.L., Y.Q.R., T.P., Y.Y.Z., and Z.Q.L. wrote and edited the manuscript. Z.Q.L., T.P., Y.Y.Z., and Y.Q.R. reviewed and made significant revisions to the manuscript. X.W.H., and Z.Q.L. revised and edited the manuscript. J.Q.C., Y.Y.Z., S.Y.W., Z.K.Z., P.L., Q.C., H.X., Y.H.B., A.N.Z., and S.T.L. collected and prepared the related papers. All authors read and approved the final manuscript.

## Ethics Statement

The authors have nothing to report.

## Consent

The authors have nothing to report.

## Conflicts of Interest

The authors declare no conflicts of interest.

## Data Availability

The authors have nothing to report.
